# Burmese injecting drug users in Yunnan play a pivotal role in the cross-border transmission of HIV-1 in the China-Myanmar border region

**DOI:** 10.1080/21505594.2018.1496777

**Published:** 2018-08-01

**Authors:** Xin Chen, Yan-Heng Zhou, Mei Ye, Yu Wang, Lin Duo, Wei Pang, Chiyu Zhang, Yong-Tang Zheng

**Affiliations:** a Key Laboratory of Animal Models and Human Disease Mechanisms of the Chinese Academy of Sciences/Key Laboratory of Bioactive Peptides of Yunnan Province, National Kunming High Level Biosafety Research Center for Non-human Primate, Kunming Institute of Zoology, Chinese Academy of Sciences, Kunming, China; b College of Life Sciences, Yan’an University, Yan’an, China; c KIZ-SU Joint Laboratory of Animal Models and Drug Development, College of Pharmaceutical Sciences, Soochow University, Suzhou, China; d Section of Science and Education, The Second People’s Hospital of Yunnan Province, Kunming, China; e Pathogen Discovery and Evolution Unit, Pathogen Discovery and Big Data Center, CAS Key Laboratory of Molecular Virology & Immunology, Institut Pasteur of Shanghai, Chinese Academy of Sciences, Shanghai, China

**Keywords:** HIV-1, IDUs; cross-border, transmission; epidemiology, China, Myanmar

## Abstract

Injecting drug users (IDUs) are the major risk group for HIV-1 infection in the China-Myanmar border area. There are a large number of Burmese IDUs living in Yunnan (Yunnan-mIDUs) who might be associated with the cross-border transmission of HIV-1. From 2010 to 2013, 617 Yunnan-mIDUs were recruited from three counties of Yunnan, 19.0% of whom were detected to be HIV-1 positive by serological testing. Partial HIV-1 *p17, pol, vif-env*, and *env* genes were amplified from the positive samples and were sequenced. Phylogenetic and HIV-1 subtyping analyses revealed that HIV-1 recombinant forms (RFs), including RF_BC (36.4%), RF_01BC (26.1%), RF_01C (9.1%) and RF_01B (1.1%), were predominant among this cohort. Of the identified HIV-1 strains, 14.8%, 9.1% and 3.4% belonged to subtype C, CRF01_AE and subtype B, respectively. Transmission cluster analysis showed that sequences from the Yunnan-mIDUs formed transmission clusters not only with those from Burmese IDUs but also with those from Chinese IDUs, indicating that Yunnan-mIDUs might acquire HIV-1 infection from or spread HIV-1 to both Burmese and Chinese IDUs. Phylogeographic analyses revealed three cross-border transmission patterns associated with Yunnan-mIDUs, in which Yunnan-mIDUs served as the crucial nodes linking the Burmese and Chinese IDUs. These results suggest that Yunnan-mIDUs are a potential viral reservoir for the diffusion of HIV-1 in Yunnan and play a pivotal role in the bidirectional cross-border transmission of HIV-1 in the China-Myanmar border region. More intervention efforts that focus on Yunnan-mIDUs are recommended in Yunnan’s campaign against HIV/AIDS.

## Introduction

The China-Myanmar border region is adjacent to the “Golden Triangle,” the main opium-production area in Southeast Asia. This region is an important interchange station for the transport of drugs. A large number of injecting drug users (IDUs) are active in this region and are associated with the cross-border transmission of human immunodeficiency virus type 1 (HIV-1) [1]. In the 1980s, the Thailand-original subtype B (Thai-B or B’), circulating recombinant form (CRF) 01_AE, and the India-original subtype C were introduced into this region, which led to the co-circulation of several HIV subtypes and to the ongoing generation of numerous recombinant HIV-1 strains [2–6]. Fourteen new CRFs have been identified in this region, including the two strains circulating nationwide in China, CRF07_BC and CRF08_BC [7–12].

HIV-1 prevalence among IDUs in Yunnan (especially in the China-Myanmar border region) were 18.3%, much higher than the national prevalence of both Myanmar (0.6%) and China (< 0.1%) [13,14]. Injection drug use and needle-sharing were the most predominant risk factors for the spread of HIV-1 in this region and were responsible for the rapidly increased genetic diversity of HIV-1 by inter-subtype recombination. HIV-1 cross-border transmission in the China-Myanmar border region was closely associated with drug trafficking routes, and had evolved from single-direction dissemination (Myanmar-to-China) to bidirectional cross-border dissemination between Myanmar and China [1,15]. HIV Recombinant forms (RFs), including unique recombinant forms (URFs) and CRFs, were predominant in the China-Myanmar border region [2–6]. In particular, the proportions of RF_BC (82.4% vs. 48.1%) and RF_01BC (3.3% vs. 36.7%) among IDUs were markedly different between the Yunnan and Myanmar sides, respectively [3–5]. This difference might imply that there were specific populations responsible for HIV-1 cross-border dissemination.

We previously demonstrated that Burmese long-distance truck drivers, a high-risk group for HIV-1 infection, were associated with the cross-border transmission of HIV-1 and were linked with high-risk groups of IDUs and female sex workers [2]. IDUs are believed to be associated with HIV-1 cross-border dissemination in this region; however, the means by which they are involved in the process needs to be further determined.

A large number of cross-border migrants work, live or travel in Yunnan. In the first half of 2016, approximately 14 million border crossings took place in land ports of the China-Myanmar border region. To achieve the UNAIDS 90–90–90 goals, a series of harm reduction programs that focused on foreigners were performed by the Yunnan government [16]. From January 2003 to December 2012, the HIV status of over 70,000 foreigners entering Yunnan from Myanmar was detected, and 1961 were found to be HIV-1 seropositive [17]. Compared with those (e.g., businessmen and travelers) who crossed the border through land ports, transnational migrant IDUs more commonly went through convenient byways along the 2,185-kilometer border between China and Myanmar for the purpose of purchasing drugs, visiting family or friends, and/or doing business or odd jobs [18,19]. These travelers more easily evaded border surveillance and HIV monitoring. Furthermore, Burmese IDUs in Yunnan (Yunnan-mIDUs) preferred to inject drugs together [20]. These observations suggest that Yunnan-mIDUs might be an important high-risk population for HIV-1 cross-border dissemination in the China-Myanmar border region.

**Figure 1.**
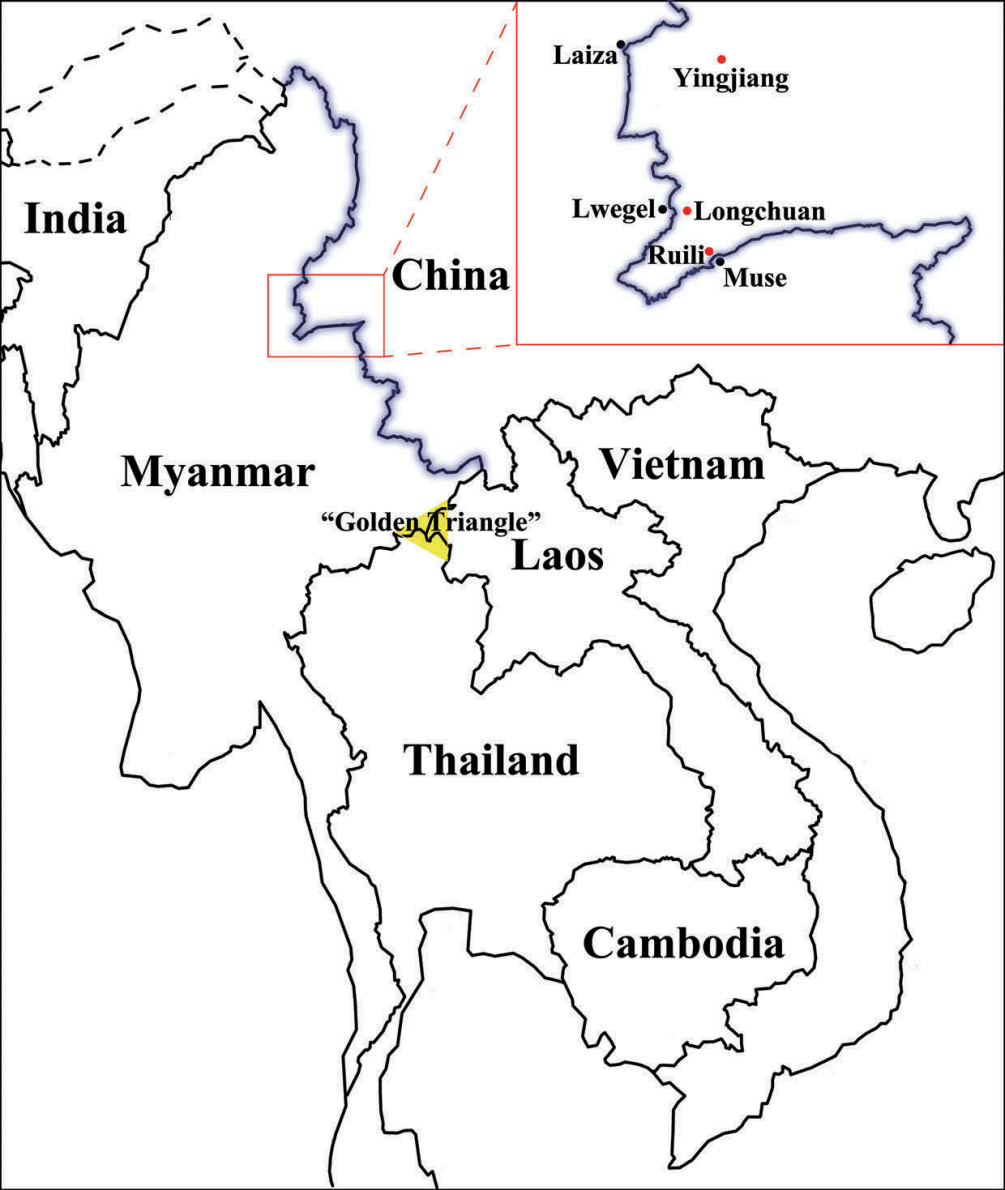
The geographic location of the China-Myanmar border region and sample sites. The black line with a blue shadow shows the border between China and Myanmar; the red and black spots indicate sample sites in China and their border regions in Myanmar, respectively; and the yellow triangle indicates the well-known illegal drug production region called the “Golden Triangle.”.

In this study, we recruited 617 Yunnan-mIDUs through a cross-sectional survey in three counties near the border (Figure 1) and investigated the molecular epidemiology and transmission dynamics of HIV-1 based on multiple-genomic fragment analysis. Our results highlighted a pivotal role played by Yunnan-mIDUs in the cross-border dissemination of HIV-1 in the China-Myanmar border region.

## Results

### Demographic characteristics and HIV-1 prevalence

A total of 617 Yunnan-mIDUs were recruited with questionnaires and blood collections (Table 1). Most of them were male (99.7%), farmers (81.4%) and 20–40 years old (77.2%) and had less than six years’ education (79.7%). About half were married (51.3%). Of the participants, 27.6%, 27.0% and 24.1% belonged to the Han, Jingpo and Dai ethnic groups, respectively.

**Table 1.** The basic data for the samples from Burmese injecting drug users in Yunnan, China.

Sample county	Bordering county of Myanmar	Sample month	Total samples	HIV-1 positive samples
Longchuan	Lwegel	2010.1–2012.12	322	51(15.8%)
Yingjiang	Laiza	2012.2	200	39(19.5%)
Ruili	Muse	2013.3–2013.6	95	27(28.4%)

The HIV-1 seroprevalence among the Yunnan-mIDUs was 19.0% (117/617). Ruili had the highest HIV prevalence (28.4%), followed by Yingjiang (19.5%) and Longchuan (15.8%, Table 1). The HIV-1 prevalence among the Yunnan-mIDUs appeared to be statistically distinct in the three counties (*P *= 0.022, chi-square test), even though different times of sampling may slightly affect the seropositive rates. There were no significant differences in HIV-1 prevalence between the different subcategories of gender, age, ethnicity, occupation, educational level and marital status.

### HIV-1 subtyping

**Figure 2.**
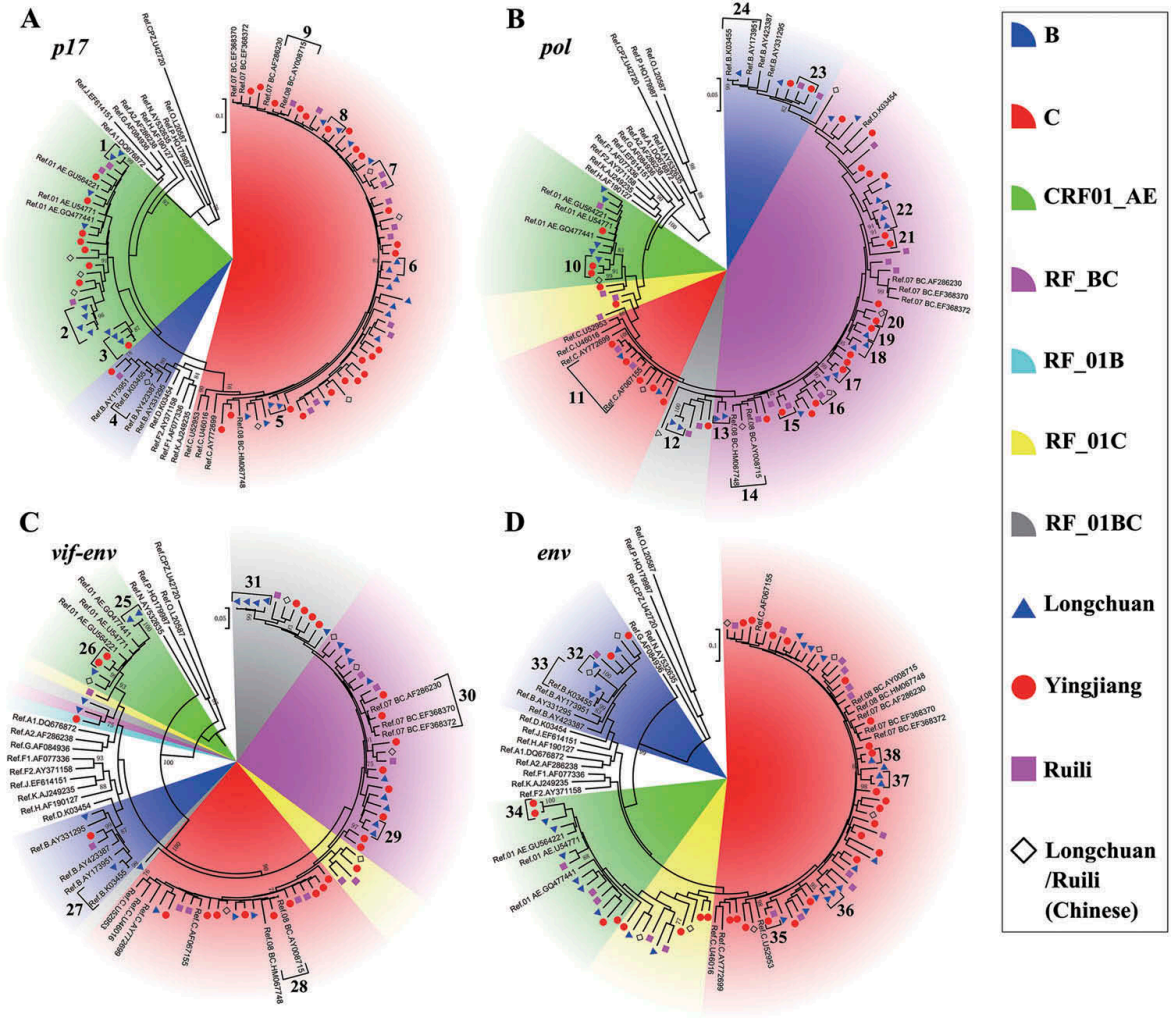
Maximum likelihood trees and cluster analysis results for the HIV-1 *p17, pol, vif-env* and *env* fragments sampled from Burmese injecting drug users in Yunnan, China. The sectors with different colors indicate different subtypes/recombinant forms of HIV-1; the blue triangles, red spots and purple squares indicate the sequences that were sampled in Longchuan, Yingjiang and Ruili Counties of Dehong Prefecture among Burmese injecting drug users, respectively; the black rhombuses indicate the sequences that were sampled in Longchuan and Ruili Counties of Dehong Prefecture among Chinese injecting drug users; and the brackets with numbers indicate transmission clusters that were identified by Cluster Picker.

Using plasma, *p17, pol, vif-env* and/or *env* genes were successfully amplified from 100 HIV-1 positive samples, with a success rate of 85.5%. The maximum likelihood (ML) trees for the *p17* and *env* fragments showed that these samples were predominantly subtype C, followed by CRF01_AE (Figure 2). For the *pol* and *vif-env* fragments, recombinant forms derived from HIV-1 subtypes B and C (RF_BC) were predominant (Figure 2).

Combining the results from the *p17, pol, vif-env* and *env* fragments, HIV-1 RFs accounted for 72.7% of the cases, showing a predominant place among Yunnan-mIDUs. The RFs covered all the possible recombinant patterns between subtypes B, C, and CRF01_AE. RF_BC, RF_01BC, RF_01C and RF_01B accounted for 36.4%, 26.1%, 14.8% and 1.1% of the cases, respectively. Subtypes C, CRF01_AE and B accounted for 14.8%, 9.1% and 3.4% of the cases, respectively.

### Transmission network

To explore the genetic and transmission relationships between Yunnan-mIDUs and Burmese and Chinese IDUs, cluster analyses were performed. A total of 820 sequences with the highest similarity to the query sequences were downloaded from the Los Alamos National Laboratory HIV-1 sequence database (HIVDB), were used in the analyses with the sequences obtained from this study (Table 2). The downloaded sequences included 363 sequences from China (54.6%), 107 from Myanmar (16.1%), 85 from Thailand (12.8%) and 39 from India (5.9%), and were available from the authors on request. The analyses of *p17, pol, vif-env* and *env* identified 48, 45, 21 and 28 transmission clusters, respectively (Table 2). The transmission clusters were more easily found in the *p17* and *pol* segments than in the *vif-env* and *env* segments, as the former two fragments were more commonly used for HIV-1 subtyping and transmission cluster analysis [21–24]. For the *pol* gene, the most frequently observed transmission clusters were formed from sequences from the Yunnan-mIDUs and Chinese IDUs, accounting for 37.8% of the clusters. Sequences from the Yunnan-mIDUs appeared to more frequently form transmission clusters with sequences from the Chinese IDUs than with sequences from the Burmese IDUs (*P *< 0.001, Fisher’s exact test), suggesting a potential networking between Yunnan-mIDUs and Chinese IDUs. Importantly, seven (15.6%) transmission clusters consisted of sequences from the Yunnan-mIDUs, Chinese IDUs and Burmese IDUs, while only three (6.7%) clusters consisted exclusively of Chinese and Burmese IDUs sequences (*P *= 0.179, Fisher’s exact test). Similar results were observed in the *p17* gene analysis (Table 2)

**Table 2.** Cluster analysis results for HIV-1 fragments sampled from Burmese injecting drug users in Yunnan, China.

				Transmission cluster								
HIV-1 fragment	Amplified sequences	Fragment size (bp)	Similar sequences^a^	CN	MM	Ym	CN+ MM	Ym+ CN	Ym+ MM	Ym+ CN+ MM	Total	Sample year ^c^
*p17*	75	573	211	10	1	3	2	22	7	3	48	1996–2013
*pol*	73	1316	181	8	1	3	3	17	6	7	45	1997–2013
*vif-env*	63	1401	141	11	1	3	0	3	2	1	21	1997–2012
*env*	77	566	191	19	0	6	1	2	0	0	28	1997–2012
Total	316	3856	820	48	3	15	6	44	15	11	142	1996–2013

a: Similar sequences were blasted using the Los Alamos HIV Sequence Database online tool, the 10 top matches were downloaded, and sequences with a genetic distance of less than 2% were removed.

b: Ym indicates that the clustered sequences were sampled from Burmese injecting drug users in Yunnan, China; CN and MM indicate that the clustered sequences were sampled from individuals in China and Myanmar, respectively.

c: The earliest and latest year in which the clustered sequences were sampled is presented.

### Role of Yunnan-mIDUs in HIV-1 cross-border transmission

**Figure 3.**
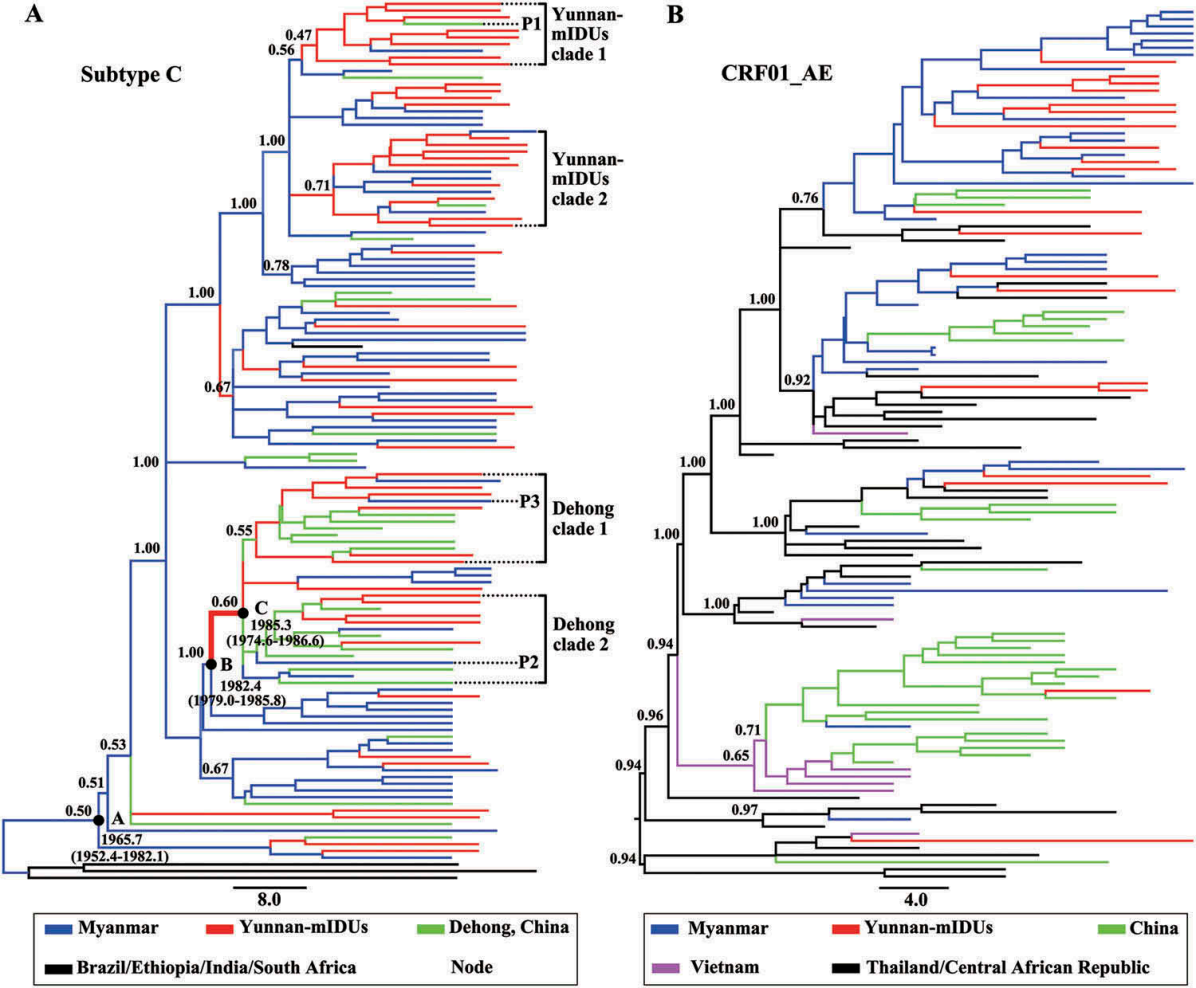
The maximum clade credibility tree based on the subtype C and CRF01_AE sequences of the HIV-1 *p17* fragment in the China-Myanmar border region. The different colored lines indicate the HIV-1 strains from different geographical locations, and the black spots indicate the nodes of HIV-1 lineages. The state posterior probabilities are indicated beside major nodes, and the ages of nodes A, B and C are shown with 95% confidence interval.

To reveal the role of Yunnan-mIDUs in the cross-border transmission of HIV-1, we investigated the genetic relationship of HIV-1 strains from the Yunnan-mIDUs, Burmese IDUs and Chinese IDUs by Bayesian phylogeographic analyses of HIV-1 *p17* fragments with subtype C and CRF01_AE origins. The maximum clade credibility (MCC) tree for subtype C (Figure 3(a)) showed that all HIV-1 strains from the China-Myanmar border region had a most recent common ancestor with a geographic origin in Myanmar in the year 1965.7 (node A). HIV-1 strains from the Yunnan-mIDUs were dispersed in the clade of the China-Myanmar border region, and clustered with those from Burmese IDUs or Chinese IDUs. HIV-1 transmission from Burmese IDUs to Yunnan-mIDUs and Chinese IDUs was often observed. Interestingly, we found two clades formed mainly from HIV-1 strains from Yunnan-mIDUs (Yunnan-mIDUs clades). These two clades had a geographic origin in Myanmar, indicating that these Yunnan-mIDUs acquired HIV-1 infection from Burmese IDUs. In the Yunnan-mIDUs clades, we observed HIV-1 transmission from Yunnan-mIDUs to Burmese IDUs, as well as to Chinese IDUs. The observation of HIV-1 transmission from Yunnan-mIDUs to Burmese IDUs was not surprising, while the observation of HIV-1 transmission from Yunnan-mIDUs to Chinese IDUs implied that Yunnan-mIDUs might serve as hubs for the cross-border transmission of HIV-1 from Myanmar to China.

We found two clades with strains from Chinese IDUs (Dehong clades) in root positions. Interestingly, the common ancestor (node C) of the Dehong clades was inferred to be a Yunnan-mIDU, and the time frame was estimated to be 1982.4–1985.3. These observations further supported the mediation of HIV-1 transmission from Myanmar to Yunnan (node B to C) by Yunnan-mIDUs. In particular, HIV-1 transmission from Chinese IDUs to Yunnan-mIDUs and then to Burmese IDUs was also observed in this clade. These results suggested that Yunnan-mIDUs mediated the reverse transmission of HIV-1 from Yunnan to Myanmar, which was different from the early Myanmar-to-Yunnan transmission.

We further analyzed the phylogeny and transmission of HIV-1 CRF01_AE *p17* fragments (Figure 3(b)). Two main transmission routes of CRF01_AE were observed. One route was from Thailand to Myanmar to China, and another route was from Thailand to Vietnam to China. Almost all HIV-1 strains among the Yunnan-mIDUs originated from Burmese IDUs except one strain that originated from Chinese IDUs. No further HIV-1 transmission mediated by Yunnan-mIDUs was observed, possibly because the involvement of CRF01_AE in the unique recombinant forms in the China-Myanmar border area was relatively later than that of subtypes B and C.

**Figure 4.**
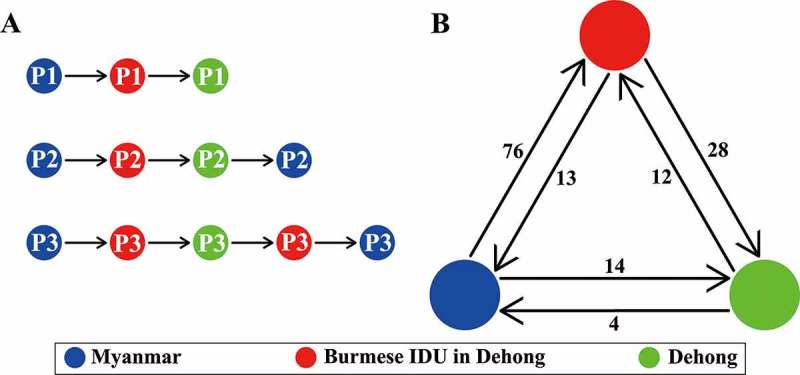
Schematic diagram of HIV-1 transmission patterns and trends in the China-Myanmar border region. The different colored spots indicate the HIV-1 strains from different geographical locations; the arrows indicate the transmitted directions; and the numbers beside them show the routings (a) or sequences (b) of each transmission direction.

To highlight the role of Yunnan-mIDUs in the cross-border transmission of HIV-1, we summarized the transmission patterns and counted the transmission events of the subtype C fragment (Figure 4). Three patterns were observed, including Burmese IDUs to Yunnan-mIDUs to Chinese IDUs, Burmese IDUs to Yunnan-mIDUs to Chinese IDUs to Burmese IDUs, and Burmese IDUs to Yunnan-mIDUs to Chinese IDUs to Yunnan-mIDUs to Burmese IDUs. These results indicated that Yunnan-mIDUs were involved in the spread of HIV-1 between Myanmar and Yunnan. The statistics for the transmission events showed that Yunnan-mIDUs more likely acquired HIV-1 infection from Burmese IDUs than from Chinese IDUs (76 vs. 12 events, *P *< 0.001, Fisher’s exact test), and more likely spread HIV-1 to Chinese IDUs than to Burmese IDUs (28 vs. 13 events, *P *= 0.002, Fisher’s exact test). These results suggested that Yunnan-mIDUs more likely participated in the spread of HIV-1 from Myanmar to China (Yunnan) than in the reverse transmission from Yunnan to Myanmar.

## Discussion

IDUs in the China-Myanmar border region is a special risk group for HIV-1 infection as they can readily migrate across the border and stay as long as years in the other side, having more opportunities for contact with both Burmese and Chinese IDUs [18,19]. From 2010 to 2013, we recruited 617 Yunnan-mIDUs in three counties of Yunnan Province. We found that the prevalence of HIV-1 among these Yunnan-mIDUs was 19.0%. The phylogenetic analysis revealed that the predominant HIV-1 strains circulating in this cohort were RFs. The transmission cluster and phylogeographic analyses revealed that Yunnan-mIDUs played a pivotal role in the cross-border transmission of HIV-1 in the China-Myanmar border region. This was the first study to reveal the role of Yunnan-mIDUs in the cross-border transmission of HIV-1.

Since the late 20th century, China has made vigorous responses to the HIV/AIDS epidemic, including implementing the “Four Frees and One Care” policy and 5-year action plans, as well as issuing a series of regulations for the prevention and control of HIV/AIDS [25–28]. Through a series of intervention efforts such as needle exchange, condom distribution and knowledge education, the prevalence of HIV-1 among IDUs in Yunnan dropped from 79.7% in 1991 to 18.3% in 2012 [13,29]. However, the regions hit hardest by HIV/AIDS in Yunnan were located in the border regions, especially in the China-Myanmar border region, where a large number of migrants and IDUs from Myanmar gathered. From 2003 to 2012, 22,699 individuals who entered Yunnan from Myanmar via the land port in Dehong Prefecture were investigated, 5.12% of whom were identified to be HIV-1 seropositive [30]. Although the HIV prevalence among Chinese IDUs in the China-Myanmar border region was controlled at a relatively low level (8.9%), Burmese IDUs in Northern Myanmar still maintained a high HIV prevalence (13.1%) [5,31]. In this study, we revealed that the HIV-1 prevalence among migrant IDUs (Yunnan-mIDUs) was 19.0%, similar to the prevalence found in previous studies [13,32]. These results imply that migrant IDUs in the border region might be a great burden for Yunnan in the control of HIV/AIDS.

Conversely, the HIV-1 strains circulating in the China-Myanmar border region showed a rapidly increased genetic diversity [3,5,6,33]. Pure HIV-1 subtypes, including B, C and CRF01_AE, were rarely detected among IDUs in this region; this increase in genetic diversity was accompanied by the rapidly increased prevalence of HIV-1 inter-subtype recombinants (greater than 87.3%) [3,4]. The most common HIV-1 recombinants were RF_BC and RF_01BC. We found that the proportions of RF_BC and RF_01BC were 36.4% and 26.1%, respectively, among the Yunnan-mIDUs; these values were intermediate between those among the IDUs in Dehong (82.4% and 3.3%) and Northern Myanmar (48.1% and 36.7%) [3,4]. Therefore, Yunnan-mIDUs were hypothesized to mediate HIV-1 cross-border transmission through contact with both Burmese and Chinese IDUs. This hypothesis was supported by the transmission cluster and phylogeographic analyses (Table 2, Figure 3). Yunnan-mIDUs could acquire HIV-1 infection from Burmese IDUs in Myanmar or from Chinese IDUs in Dehong and could spread HIV-1 to Chinese IDUs or Burmese IDUs. Yunnan-mIDUs appeared to be the crucial nodes linking Burmese and Chinese IDUs (Figure 4(a)), and they played a pivotal role in HIV-1 transmission from Myanmar to Yunnan, China, and from Yunnan to Myanmar (Figure 3) [1,12,15].

Transmission cluster analyses based on the *pol* gene showed that the sequences from the Yunnan-mIDUs more likely formed transmission clusters with those from the Chinese IDUs (Table 2). However, in our previous study that focused on HIV-1-negative but HCV-positive IDUs, we found that HCV sequences from Yunnan-mIDUs preferentially formed transmission clusters with themselves instead of with HCV sequences from Chinese IDUs [20]. HIV and HCV share the same transmission routes. Almost all HIV-positive IDUs were HCV-positive, while approximately 70% of the HCV-positive IDUs were HIV-positive [32,34]. Although it was unclear whether the differences in the transmission patterns of HCV and HIV-1 reflected different stages of Yunnan-mIDUs’ stay in Yunnan, Yunnan-mIDUs were believed to preferentially form a relatively isolated independent group of injecting drug users separate from Chinese IDUs, possibly because of the unfamiliar environment and lack of interpersonal relationships at early stages of their stay in Yunnan. Once they integrated into the local community of IDUs, HIV-positive individuals might spread HIV-1 to local IDUs, and HIV-negative individuals might acquire HIV-1 infection and further spread the virus to their Burmese partners (IDUs or sexual partners). Therefore, the sociological, psychological and behavioral characteristics of Yunnan-mIDUs deserve further investigation.

Yunnan-mIDUs had a relatively higher prevalence of HIV-1 and could easily cross the China-Myanmar border through land ports and convenient routes [18,19]. These factors implied a high possibility of HIV-positive Burmese IDUs entering Yunnan. In addition, non-Chinese immigrants, especially migrant IDUs, had very low rates of HIV-1 test, antiretroviral treatment and viral suppression in Yunnan, and these immigrants may serve as a potential virus reservoir for the diffusion of HIV-1 in Yunnan [35]. Our recent study showed that HIV-positive IDUs were a crucial population in which to reduce needle-sharing behavior and HIV infection [36]. Therefore, more attention should be paid to Yunnan-mIDUs by the governments of both China and Myanmar and some non-governmental organizations to reduce the HIV-1 transmission associated behaviors and to monitor the molecular epidemiological characteristics of HIV-1 among this risk group [35].

There are two limitations in the interpretation of the results of this study. First, the sample site was limited to Dehong Prefecture of Yunnan Province. Seven prefectures border other countries in Yunnan, and Dehong is the region most drastically impacted by HIV-1. The results obtained in Dehong may be an archetype of those in other border prefectures of Yunnan Province. Second, a limited number of HIV-1 sequences were obtained from Myanmar; this factor may influence the exact number of identified clusters for each cross-border transmission pattern. Despite these limitations, our results showed a clear map of HIV-1 transmission dynamics in the China-Myanmar border region and provided valuable information for harm reduction programs related to HIV/AIDS control.

In summary, we reported an HIV-1 prevalence of 19.0% among Yunnan-mIDUs from 2010 to 2013 and revealed that HIV-1 recombinants were the predominant strains in this cohort. In particular, we found that Yunnan-mIDUs had a close transmission relationship with both Burmese and Chinese IDUs, which highlighted the pivotal role of Yunnan-mIDUs in the bidirectional cross-border transmission of HIV-1 in the China-Myanmar border region. More intervention efforts with Yunnan-mIDUs should be conducted in Yunnan’s campaign against HIV/AIDS to achieve the UNAIDS 90-90-90 goals.

## Materials and methods

### Ethics statement

This study was approved by the Ethics Committee of Kunming Institute of Zoology, Chinese Academy of Sciences (approval number: SWYX-2009021; approval date: 7 January 2009). Written informed consent was obtained from all participants.

### Participants

Under the support of the HIV/AIDS Asia Regional Program Yunnan Management Office and local governments, a cross-sectional survey was carried out among Yunnan-mIDUs in communities and detoxification centers of Longchuan, Yingjiang and Ruili Counties, Dehong Prefecture, Yunnan Province, China, between 2010 and 2013 (Figure 1, Table 1). To be eligible, survey participants in this study must have satisfied the following requirements: 1) Burmese nationality, 2) at least 18 years old, and 3) history of injected drug use in the past six months.

With the help of trained native outreach workers, a face-to-face interview was conducted with a standard questionnaire regarding baseline sociodemographic information. Blood samples were collected and then centrifuged. The serum was stored in a −80°C freezer prior to the following HIV testing and gene fragment amplification experiments.

### HIV testing

The HIV serostatus of Yunnan-mIDUs was assessed with the Alere Determine™ HIV-1/2 Ag/Ab Combo (Alere Medical Co., Ltd., Tokyo, 7D2649) and was confirmed by the Diagnostic Kit for Antibodies to the Human Immunodeficiency Virus (Beijing Wantai Biological Pharmacy Enterprise Co., Ltd., Beijing), according to the manufacturer’s instructions.

### HIV-1 sequence generation

For the HIV-1 seropositive samples, partial HIV-1 *p17, pol, vif-env* and *env* genes were amplified as previously described [3]. Briefly, viral RNA was extracted from plasma using the High Pure Viral RNA Kit (Roche Diagnostics Ltd., Mannheim, 11858882001). cDNA was synthesized using the PrimeScript II 1st Strand cDNA Synthesis Kit (TaKaRa Biomedical Technology Co., Ltd., Beijing, 6210A). HIV-1 fragments were amplified with nested PCR using the TransTaq DNA Polymerase High Fidelity kit (Beijing TransGen Biotech Co., Ltd., Beijing, AP131-13). The nested PCR products were purified using a Gel Extraction Kit (Bioteke Corporation, Beijing, DP1503) and were sequenced with an ABI PRISM 377XL DNA sequencer (Applied BioSystems, California).

For the purpose of exploring the transmission relationship between Yunnan-mIDUs and Yunnan IDUs, the partial HIV-1 *p17, pol, vif-env* and *env* genes of 11 Chinese IDUs who were recruited in the same cross-sectional survey were amplified with the methods described above.

### Phylogenetic analysis

To discern the subtypes of the amplified sequences, reference sequences relevant to Southeast Asia were downloaded from HIVDB. After hand-curating to ensure the correct open reading frame, the query sequences were aligned with the reference sequences. ML trees were constructed using MEGA7.0 with 1000 bootstrap replications. For the sequences that did not cluster with reference sequences, bootscan analysis was performed to identify potential HIV-1 recombinants using Simplot 3.5.1, as previously described [5].

### Cluster analysis

To explore the potential transmission linkage between Yunnan-mIDUs and Chinese IDUs in Dehong, transmission clusters of the HIV-1 sequences were identified using Cluster Picker 1.2.4 [37]. According to the Cluster Picker manual and the suggestions of previous studies, the parameter support threshold was set to 0 and the genetic distance threshold was set to 4% to identify a close transmission relationship between Yunnan-mIDUs and Dehong IDUs [22–24,38].

As a limited number of sequences were amplified in this study, to further explore the HIV-1 transmission networks related to Yunnan-mIDUs, the sequences were blasted in the HIVDB and the top 10 matches of each sequence were downloaded. Sequences with a genetic distance of less than 2% were eliminated to minimize the calculations for generating the ML trees. After alignment with the subtype reference sequences, ML trees were constructed and transmission clusters were identified, as described above.

### Phylogeographic analysis

HIV-1 has a high mutation rate and a high potential for recombination. The *p17* gene is commonly used for phylogeographic analysis, because it is relatively more conserved than other gene regions, and studies showed that the phylogeographic result based on it is well consistent with those of other fragments, if no recombinant sequences were used [2,9,11,15]. For the purpose of investigating the role that Yunnan-mIDUs play in the cross-border transmission of HIV-1 in the China-Myanmar border region, all the *p17* sequences which originated from Myanmar or from Dehong Prefecture of China were downloaded from the HIVDB. Subsequently, the subtype C and CRF01_AE sequences were identified according to the results of both the neighbor-joining tree and the “Recombinant Identification Program” online tool available in the HIVDB. Bayesian phylogeographic analysis was performed using the BEAST v1.6.1 package. Briefly, the sampling time and geographic location of each sequence and the parameters for constructing the maximum clade credibility (MCC) trees were assigned using BEAUti v1.6.1. The evolutionary rates and the time to most recent common ancestor of each node were inferred using BEAST v1.6.1. The MCC trees were summarized with Tree Annotator v1.6.1 and were visualized using FigTree v1.4.2. The convergence and effective sample size were checked with Tracer v1.6. Other parameters have previously been described in detail [10,15].

To further explore the role of Yunnan-mIDUs in the cross-border transmission of HIV-1, the transmission patterns associated with Yunnan-mIDUs were summarized from the MCC tree of HIV-1 subtype C fragments. Additionally, the transmission events between Yunnan-mIDUs, Burmese and Chinese individuals were counted. Fisher’s exact test was used to compare the proportion of these different transmission directions.

### Sequence data

The sequences obtained in this study have been submitted to GenBank with accession numbers KX988316 to KX988415 for the sequences from the Yunnan-mIDUs, and MF000419 to MF000429 for the sequences from the Chinese IDUs.
